# M3 Receptor Pathway Stimulates Rapid Transcription of the CB1 Receptor Activation through Calcium Signalling and the CNR1 Gene Promoter

**DOI:** 10.3390/ijms24021308

**Published:** 2023-01-09

**Authors:** Pietro Marini, Philip Cowie, Ahmet Ayar, Guy S. Bewick, John Barrow, Roger G. Pertwee, Alasdair MacKenzie, Paolo Tucci

**Affiliations:** 1 Institute of Education in Healthcare and Medical Sciences, Foresterhill, University of Aberdeen, Aberdeen AB25 2ZD, UK; 2 The Institute of Medical Sciences, Foresterhill, University of Aberdeen, Aberdeen AB25 2ZD, UK; 3 Department of Physiology, Faculty of Medicine, Karadeniz Technical University, 61080 Trabzon, Turkey; 4 Department of Clinical and Experimental Medicine, University of Foggia, 71122 Foggia, Italy

**Keywords:** intracellular calcium, M_3_ receptor, CB_1_ receptor, CNR1 promoter, CREB

## Abstract

In this study, we have investigated a possible mechanism that enables CB_1_/M_3_ receptor cross-talk, using SH-SY5Y cells as a model system. Our results show that M_3_ receptor activation initiates signaling that rapidly upregulates the CNR1 gene, resulting in a greatly potentiated CB_1_ receptor response to agonists. Calcium homeostasis plays an essential intermediary role in this functional CB_1_/M_3_ receptor cross-talk. We show that M_3_ receptor-triggered calcium release greatly increases CB_1_ receptor expression via both transcriptional and translational activity, by enhancing CNR1 promoter activity. The co-expression of M_3_ and CB_1_ receptors in brain areas such as the nucleus accumbens and amygdala support the hypothesis that the altered synaptic plasticity observed after exposure to cannabinoids involves cross-talk with the M_3_ receptor subtype. In this context, M_3_ receptors and their interaction with the cannabinoid system at the transcriptional level represent a potential pharmacogenomic target not only for the develop of new drugs for addressing addiction and tolerance. but also to understand the mechanisms underpinning response stratification to cannabinoids.

## 1. Introduction

Understanding how the cascade of molecular events that follows receptor activation alters gene expression at the level of transcription, is the central pillar of the emerging field of pharmacogenomics. This is particularly important in understanding why a proportion of cannabinoid drug users suffer side effects such as addiction and withdrawal. For example, changes in intracellular Ca^2+^ concentration are essential in synaptic plasticity, such as the learning and memory processes of long-term potentiation and depression [1]. Similarly, they may have a role in the development of dependence and withdrawal associated with the abuse of drugs like cannabinoids [2]. In this context, intracellular Ca^2+^ homeostasis is crucial for the functions that neuronal systems such as the dopaminergic and glutamatergic systems display during the adaptive responses (tolerance, receptor sensitization, dependence, and withdrawal) involved in the on-set of addiction [3,4,5]. Importantly in this context, intracellular Ca^2+^ is the major intracellular messenger that links synaptic activity in neurons to gene expression, and it is involved in the regulation of signal transduction networks of G protein-coupled receptors (GPCRs), including cannabinoid receptors [6,7,8,9,10]. Recently, a role for the cholinergic system in drug abuse disorder has been emerging, with several lines of evidence demonstrating a close functional coupling between acetylcholine (ACh) and opioid transmission [11]. Similarly, the cholinergic system has been found to play a significant role in the development of addiction induced by the use of cannabinoids [12,13,14,15,16], with functional cross-talk between ACh receptors (AChRs) and the endocannabinoid system [17]. Within the cholinergic system, it has been suggested that muscarinic AChR M_3_ subtype (M_3_ receptor) play a role, although how is not well understood. So far, it has been demonstrated that activation of the M_3_ receptor, by elevating the intracellular Ca^2+^ concentration, will modulate subsequent responses to activation of cannabinoid receptor type 1 (CB_1_) and δ-opioid receptors in the same cell [10].

This modulation of pharmacological responses of CB_1_ and δ-opioid receptors observed after M_3_ receptor activation, has been generally described as a co-incident signaling pathway, resulting in the modulation of the activity of phospholipase C (PLC) and calmodulin [6,7,8,9,10]. However, it is possible that intracellular calcium may also, or instead, act by the regulation of molecular and genomic mechanisms to produce this functional cross-talk. There is an enormous impact of addiction to cannabinoids on global human health, as well as the effects of withdrawal on recovery from drug abuse more generally. It is important, therefore, to determine whether, and if so how, additional molecular and genomic mechanisms might play in modulating the cellular effects of these drugs for a holistic understanding of the obstacles to recovery from abuse. 

Most of our understanding of the functional cross-talk between M_3_ and δ-opioid or CB_1_ receptors has been elucidated through in vitro experimentation using the human neuroblastoma cell line SH-SY5Y. Amongst its many neuronal characteristics, this neuron-like cell line co-expresses both CB_1_, and M_3_ receptors, so it represents a suitable model for this kind of investigation. Indeed, we have previously established that the activities of CB_1_ and M_3_ receptors are mechanistically linked within SH-SY5Y cells, including an extensive pharmacological characterization to explain the nature of this functional cross-talk [10]. The main outcome of those studies was that CB_1_ receptors stimulate Ca^2+^ release from intracellular stores in a dose-dependent manner, but only in the presence of a concomitant M_3_ receptor activation, and PLCβ was identified as the main intracellular target involved in the mechanism [6,7,8,10]. Here, we further characterize the role of intracellular Ca^2+^ in CB_1_/M_3_ cross-talk, and whether there is a further role for any molecular and genomic mechanisms. To ensure comparability with previous studies, the receptor ligands and their dosage employed in the present investigation are the same as in our previous investigation [10]. In the present study, therefore, we have extended our investigation into the role of intracellular Ca^2+^ on the electrophysiological responses to the CB_1_ and M_3_ receptor in the SH-SY5Y cell line. Specifically, since enhancement of intracellular Ca^2+^ concentration has been demonstrated to be involved in the activation of a number of transcription factors and in the regulation of the associated genes [18,19,20,21], we have sought in the present study to determine if M_3_ activation plays a role in modulating CB_1_ expression levels, known to be critical in the proper physiological functioning of CB_1_ receptors, and specifically, in the genetic regulation of the *Cannabinoid Receptor 1* (CNR1) gene, downstream of M_3_ receptor stimulation. By providing evidence of cross-talk between M_3_ and CB_1_ receptor signalling at the transcriptional level, our study provides another stepping stone in understanding the pharmacogenomics of drug addiction and withdrawal. 

## 2. Results

### 2.1. M_3_ Receptor Activation Primes CB_1_ Receptors in Human SH-SY5Y Neuroblastoma Cells

**Figure 1:**
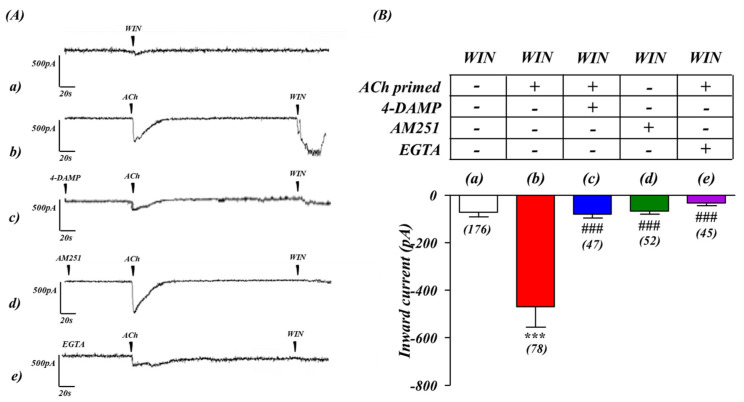
ACh ‘priming’ enhances inward current responses to CB_1_ receptor activation in SH−SY5Y human neuroblastoma cells. (**A**) Whole cell voltage-clamp recordings from SH−SY5Y cells in culture. A small inward current after CB_1_ receptor activation (WIN 55,212−2, 100 nM), was observed in SH−SY5Y cells (**a**), 5 min pre-application of acetylcholine (ACh, 1 µM) greatly enhances the inward current induced by WIN 55,212−2 stimulation (**b**). Superfusion with either M_3_ (4−DAMP, 10 nM) or CB_1_ (AM251, 1 μM) receptor antagonists (**c**,**d** respectively) were able to completely abolish the ACh priming effect on CB_1_ receptors. During superfusion with Ca^2+^-free/EGTA (100 µM) buffer, the ACh−dependent priming effect on CB_1_ receptors was again abolished (**e**). (**B**) Histogram representation of the M_3_ receptor priming on the subsequent cellular response induced by CB_1_ receptor stimulation in presence of 4−DAMP (**c**), AM251 (**d**) and EGTA (**e**). *** *p* < 0.001 vs. (**a**) WIN only, and ^###^ *p* < 0.001 vs. (**b**) ACh−WIN. One−way analysis of variance followed by Multiple Comparison Test, with Bonferroni’s correction. *n* values for each experiment are shown in brackets below each bar.

As detailed above, calcium imaging studies in human SH-SY5Y neuroblastoma cells show that CB_1_ receptor activation (via Gα_i/o_) stimulates Ca^2+^ release from intracellular stores only after concomitant M_3_ receptor activation (via Gα_q/11_) [10]. Here, we addressed the question of whether this priming by the M_3_ receptor also affects the CB_1_ receptor-driven electrophysiological response. To elucidate this, whole cell patch-clamp recordings were obtained of currents evoked by CB_1_ receptor activation alone, or after pre-exposure to ACh, from SH-SY5Y cells grown on glass coverslips. The activation of CB_1_ receptors by the localized application of the CB_1_ receptor full agonist, WIN 55,212-2 (WIN, 100 nM) ejected by pressure from the tip of a nearby pipette, produced only a small inward current when applied to naïve cells (Figure 1Aa, Ba). Conversely, the pressure application of ACh to activate M_3_ receptors (1 µM; Figure 1Ab) induced a substantial inward current. However, strikingly, if applied within 5 min after ACh application, WIN 55,212-2-induced inward currents were greatly enhanced (*p* < 0.001) (Figure 1Ab, Bb). This enhanced response was due to ACh-priming, since it was abolished either in presence of the selective M_3_ receptor antagonist 4-DAMP (10 nM, Figure 1Ac, Bc) or the selective CB_1_ receptor antagonist AM 251 (1 µM, Figure 1Ad, Bd). Thus, ACh priming, via muscarinic M_3_ receptors stimulation, induced a greatly enhanced electrophysiological response to CB_1_ receptor activation.

### 2.2. Enhanced CB_1_ Mediated Response in the Presence of ACh Is Due to the Release of Intracellular Ca^2+^

To determine if the enhanced WIN 55,212-2 response due to ACh priming required the release of Ca^2+^ from internal stores, we tested if it was abolished when recording in calcium-free extracellular buffer (0 Ca^2+^/EGTA buffer), which depletes intracellular Ca^2+^ stores. This was indeed the case, since the ACh-primed enhancement of the WIN 55,212-2 response was greatly reduced or abolished in the absence of Ca^2+^ (Figure 1Ae,Be). This indicates that the enhanced electrophysiological response to CB_1_ agonist after the ACh priming, was Ca^2+^-dependent.

### 2.3. ACh-Primed Enhancement of the CB_1_-Mediated Response Is Modulated at the Transcriptional and Translational Level 

**Figure 2:**
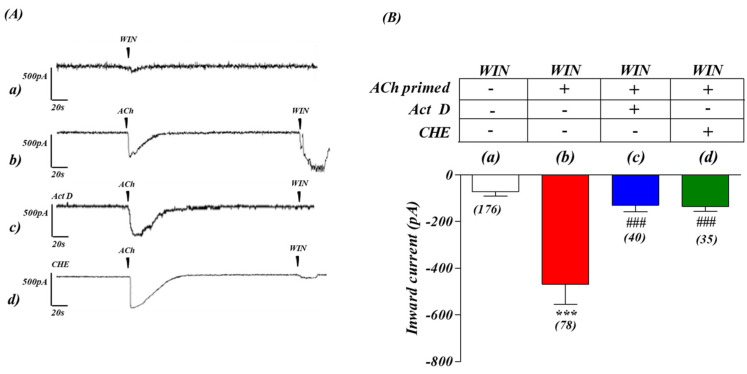
ACh ‘priming’-enhanced inward current responses to CB_1_ receptor activation in SH−SY5Y human neuroblastoma cells involves transcription and protein synthesis. (**A**) Whole cell voltage-clamp recordings from SH−SY5Y cells in culture. The basal inward current induced by WIN 55,212−2 (**a**) was greatly enhanced after 5 min pre-application of acetylcholine (ACh, 1 µM) (**b**). The ACh−dependent priming enhancement of CB_1_ receptor responses was abolished by 30 min pre-treatment with RNA polymerase inhibitor actinomycin D (Act D, 1 mM) (**c**) or with protein synthesis inhibitor cycloheximide (CHE, 10 nM) (**d**) (**B**) Histogram representation of the M_3_ receptor priming on the subsequent cellular response induced by CB_1_ receptor stimulation in presence of Act D (**c**), and CHE (**d**). *** *p* < 0.001 vs. (**a**) WIN only, and ^###^ *p* < 0.001 vs. (**b**) ACh−WIN. One−way analysis of variance followed by Multiple Comparison Test with Bonferroni’s correction. *n* values for each experiment are shown in brackets below each bar.

We next investigated the mechanism by which intracellular Ca^2+^ enhances the electrophysiological response to WIN 55,212-2. Because it has been shown that CB_1_ receptors undergo continuous and rapid replacement and degradation [22], we explored the possibility that intracellular Ca^2+^ release accelerates de novo transcription of CB_1_ receptors. We therefore tested this hypothesis by the inhibition of the cellular machinery (transcription and translation) involved in protein production, to see if it impaired the enhancing effects of ACh on CB_1_ activity. Consistent with this hypothesis, the 30 min pre-incubation of SH-SY5Y cells with actinomycin D (Act D, a RNA polymerase inhibitor, 1 mM), blocked the ACh-priming-induced response to WIN 55,212-2 (Figure 2Ac,Bc). Furthermore, when cells were pre-incubated (30 min) with cycloheximide (CHE, a protein synthesis inhibitor, 10 nM), a similar effect upon the ACh-mediated priming-induced response to a CB_1_ receptor agonist was also observed (Figure 2Ad,Bd).

These experiments suggest that the enhancement of the CB_1_-mediated response by ACh involved both transcriptional and translational mechanisms. 

**Figure 3:**
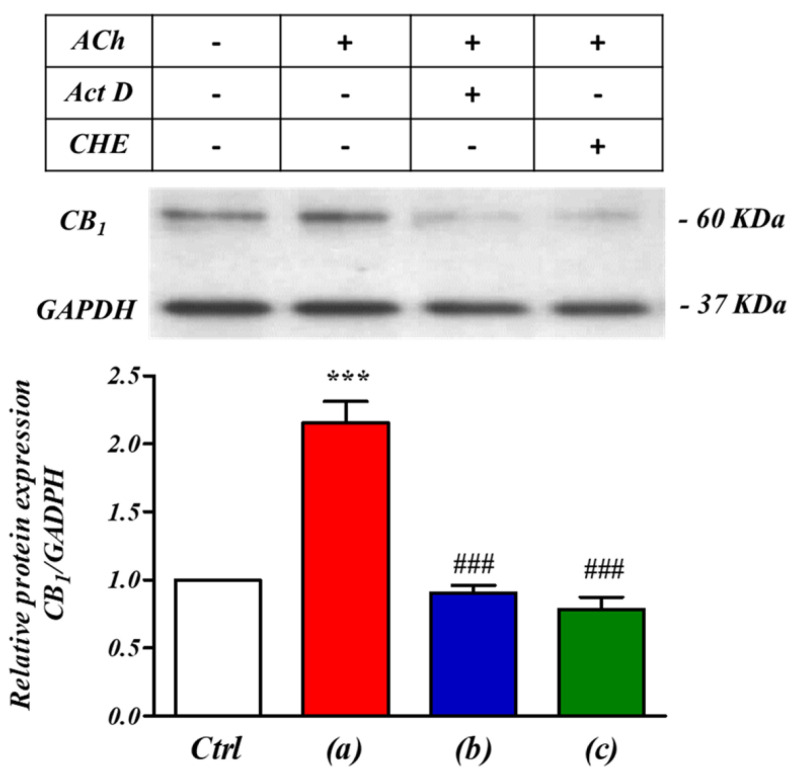
ACh ‘priming’ enhanced CB_1_ receptor expression levels through both transcriptional and translational mechanisms in SH−SY5Y human neuroblastoma cells. Western blotting analysis of endogenously expressed CB_1_ receptor levels in SH−SY5Y human neuroblastoma cells. CB_1_ receptor expression levels were significantly increased by the pre−stimulation with acetylcholine (ACh, 1 µM). This effect was abolished by pre−incubation (30 min) with the RNA polymerase inhibitor actinomycin D (Act D, 1 mM, (**b**)) or with the protein synthesis inhibitor cycloheximide (CHE, 10 nM, (**c**)). *** *p* < 0.001 vs. Ctrl, ^###^ *p* < 0.05 vs. (**a**) ACh only. One−way analysis of variance followed by Multiple Comparison Test with Bonferroni’s correction (*n* = 6).

If translational mechanisms are involved in the enhancement, Western blot analysis of SH-SY5Y cells should reveal changes in expression levels of CB_1_ receptor proteins. Indeed, 5 min of ACh stimulation (1 µM) did significantly increase expressed CB_1_ receptor protein levels (Figure 3a). To test the hypothesis that ACh application stimulated CB_1_ expression at the transcriptional level, Western blot analysis was performed on SHSY-5Y cells after treatment with the RNA polymerase inhibitor actinomycin D during ACh stimulation (Figure 3b). This prevented the increase in CB_1_ protein expression, in keeping with the hypothesis that M_3_ stimulated expression of CB_1_ receptor acted at the transcriptional level. The increased expression was also blocked when cells were incubated (30 min) with the protein synthesis inhibitor cycloheximide (10 nM) (Figure 3c). These findings both support the contention that the M_3_-induced increase of CB_1_ receptor expression is controlled at both transcriptional and translational levels.

### 2.4. CB_1_ Receptor Expression Modulated by M_3_ Receptor Stimulation Is Controlled by the CNR1 Promoter Locus

**Figure 4:**
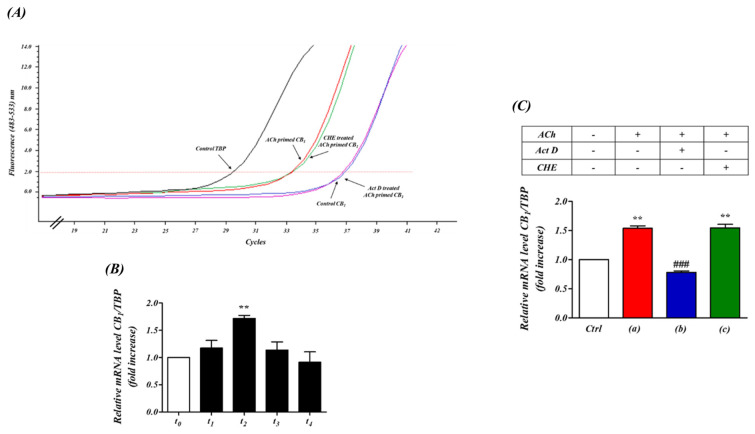
ACh ‘priming’ enhances the transcription of CB_1_ receptor acting on the CNR1 locus in SH−SY5Y human neuroblastoma cells. (**A**) qPCR amplification curves of mRNA CB_1_ receptor level (CNR1 locus) in human neuroblastoma SH−SY5Y cells. The effects of ACh, Act D and CHE on mRNA CB_1_ receptor levels are indicated by arrows. (**B**) Time course of the effect of M_3_ receptor stimulation on the mRNA CB_1_ receptor level in human neuroblastoma cell line. t_0_: 0 min; t_1_: 2.5 min; t_2_: 5 min; t_3_: 7.5 min; t_4_: 10 min (*n* = 3). ** *p* < 0.05 vs. t_0_. One-way analysis of variance followed by Multiple Comparison test with Bonferroni’s correction. (**C**) Effect of transcriptional and translation inhibitors on ACh-induced up-regulation of the CNR1 locus. The transcriptional up-regulation of the CNR1 locus was significantly induced by ACh pre-stimulation (ACh, 1 µM, 5 min, **C**(**a**)). Incubation with the RNA polymerase inhibitor actinomycin D (Act D, 1 mM, 30 min, **C**(**b**)) completely abolished this up−regulation of CNR1 transcription. In contrast, incubation with the protein synthesis inhibitor cycloheximide (CHE, 10 nM, 30 min, **C**(**c**)) had no effect on the ACh−induced up-regulation of CNR1 transcription. (TBP: TATA box binding protein). ** *p* < 0.05 vs. Ctrl, ^###^ *p* < 0.001 vs. (**A**) ACh only. One−way analysis of variance followed by Multiple Comparison test with Bonferroni’s correction. (*n* = 6).

If the enhanced response to WIN 55,212-2 via M_3_ receptor pre-stimulation is due to an increase in CB_1_ receptor mRNA expression, this should occur within the timeframe observed in the electrophysiology experiments. To examine the kinetics of these changes with more precision, we performed quantitative PCR analysis on total RNA derived from treated and untreated SH-SY5Y cells with CNR1 gene mRNA specific primers (Figure 4a). Consistent with the proposed hypothesis, the expression of the CNR1 mRNA was significantly increased by ACh stimulation with the maximum increase reached after 5 min of the cholinergic agonist administration (Figure 4b). Conversely, Act D, which inhibits transcription, abolished the ACh-induced up-regulation of CNR1 transcription (Figure 4a, c(b)). On the other hand, pre-incubation with the protein synthesis inhibitor cycloheximide, did not cause any detectable effect upon ACh-induced up-regulation of CNR1 transcription (Figure 4a, c(c)). Taken together, these results support the hypothesis that a transcriptional regulatory relationship between ACh stimulation and CNR1 transcription exists. Moreover, since in SH-SY5Y cells, ACh stimulation causes intracellular Ca^2+^ mobilization mainly through M_3_ receptors [23], these results corroborate the electrophysiological observations above showing that M_3_ receptor-mediated priming could modulate CB_1_ receptor protein levels. 

### 2.5. Genomic Annotation of the CNR1 Promoter Indicates That It Is Active in Neuroblastoma Cells, Binds Core Transcriptional Proteins and Contains Putative cAMP Response Element-Binding Protein (CREB) Binding Sites

**Figure 5:**
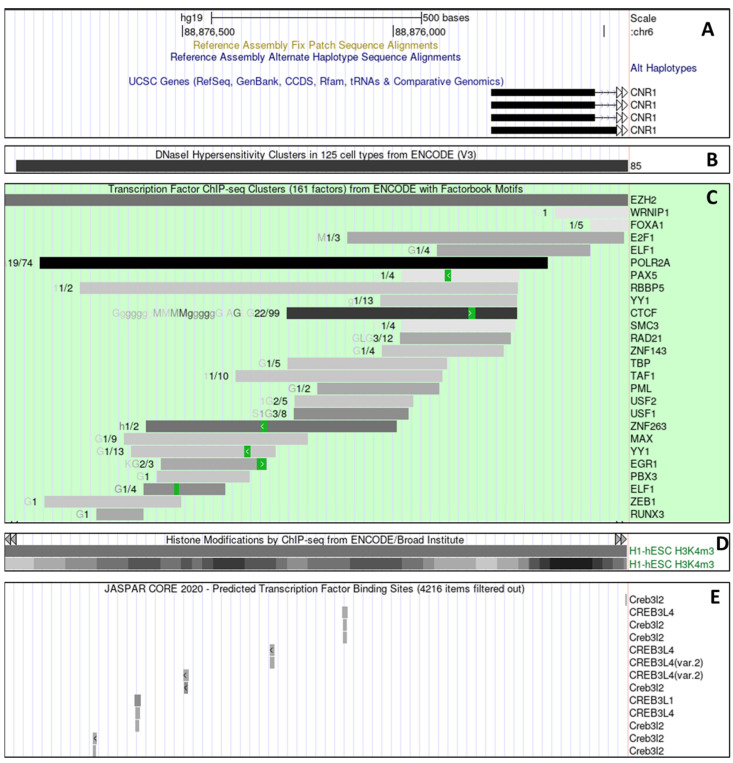
Genomic annotation of CNR1prom sequence. Genomic datamining analysis from the UCSC genome database demonstrating (**A**) 1 kb of genomic DNA 5′ of the human CNR1 locus with the CNR1 exon 1 shown as a black bar and intron 1 shown as a thin line with chevrons. (**B**) The results of DNAse1 hypersensitivity assays (Black bar) from 125 cell lines demonstrating open chromatin extending approximately 800 bp 5′ from the CNR1 transcription start site. (**C**) The results of multiple CHIP-seq analyses on different cell lines demonstrating the binding of different transcription factors and RNA polymerase II (POLR2A) to the CNR1 promoter region. (**D**) ChIP−seq analysis of modified histone (H3K4me3) highlighting promoter activity. (**E**) Predicted binding sites (Grey boxes) for the CREB transcription factor as calculated by JASPAR.

These data so far suggested that in SH-SY5Y neuroblastoma cells, ACh activation of M_3_ receptors transcriptionally regulates the CNR1 locus and its protein product, the CB_1_ receptor. The stimulation of muscarinic AChRs coupled to Gα_q/11_ (i.e., M_3_ receptors) has been shown to lead to the activation of CREB in neuronal cells, which could be how these effects occur in our experimental system [24,25]. Bioinformatics analysis was therefore conducted for the CNR1 promoter region in these cells for characteristic features of a promoter element with CREB binding sites (Figure 5). This analysis revealed that the CNR1 promoter is in an active/open conformation, consistent with its sensitivity to DNAseI digestion in a number of different cell types, including SK-N-SH cells from which SH-SY5Y cells derive (Figure 5B). Binding studies also suggest that the 5′ end of the CNR1 locus is a promoter element, as it binds RNA polymerase II (RNApol), TBP and TAF1, which are members of the transcriptional pre-initiation complex (Figure 5C). Further, the promoter region shows marked signals for H3K4me3 histone modifications, a characteristic of active promoter regions (Figure 5D). Taken together, the current genomic annotation of the 5′ region of the CNR1 locus is consistent with previous work indicating the presence of a promoter at this locus [24], i.e., functioning as a CNR1 promoter. 

Therefore, overall, these data support the hypothesis that the CNR1 promoter contains a response element that responds to signal transduction pathways triggered by activation of mAChRs, that coordinately regulate the expression of the CB_1_ receptor. Further examination of the CNR1 promoter was therefore conducted to establish if it could bind to, and regulate transcription in response to, ACh-induced CREB activation. Consistent with this, JASPAR analysis (Figure 5E) revealed numerous highly conserved putative CREB binding sites. This analysis indicates, therefore, the presence of a highly conserved mechanism by which ACh-induced transcription regulation can alter the levels of CB_1_ receptor protein by activating transcription factors such as CREB to modulate the activity of key regulatory domains of the CNR1 locus. However, it is possible that the interaction of CREB with the CNR1 promoter is indirect rather than through the direct binding of CREB to the CNR1 promoter. Further analysis of this possible interaction is ongoing.

### 2.6. The Promoter Region of CNR1 Is Sensitive to M_3_ Receptor Stimulation

**Figure 6:**
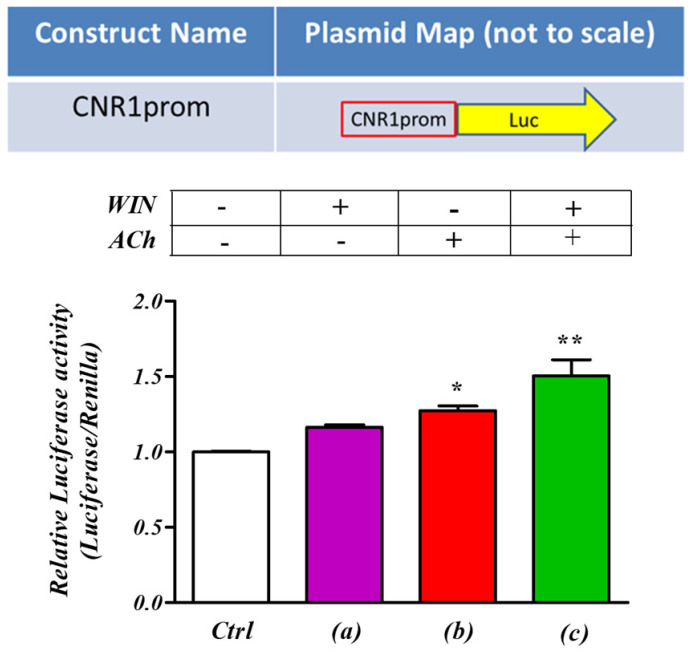
ACh ‘priming’ sensitizes the proximal promoter region of CNR1. Diagrammatic representation of the luciferase reporter construct used in the experiments with human neuroblastoma cells, SH−SY5Y. The cells were transfected with high quality pCNR1 prom reporter plasmid and treated for 30 min with the CB_1_ receptor agonist WIN 55,212-22 (WIN, 100 nM, (**a**)), or the M_3_ receptor agonist acetylcholine (ACh, 1 µM, (**b**)), or with a combination of both (**c**). Following stimulation of transfected SH−SY5Y cells with WIN 55,212-2, no significant change was seen. However, after ACh treatment, a significant increase in luciferase expression was observed. A greater magnitude of CNR1 promoter activity was observed when the transfected cells were stimulated with both CB_1_ (WIN) and M_3_ (ACh) receptor agonists. * *p* < 0.05 vs. Ctrl, ** *p* < 0.01 vs. Ctrl. One−way analysis of variance followed by Multiple Comparison test, with Bonferroni’s correction. (*n* = 6).

A number of physiological and pathological conditions involving CB_1_ receptor expression level and functionality have been related to the genetic regulation of the CNR1 promoter region [24]. The next experiment therefore tested if the previously observed M_3_ receptor-stimulated increase in CNR1 transcriptional activity was via the CNR1 promoter. A luciferase reporter plasmid was produced, containing 1 kb of the CNR1 promoter region, based on observations published by Zhang et al. [23] and information produced from our datamining (Figure 5). The reporter construct was then transfected into SH-SY5Y cells that were subsequently stimulated with ACh and the luciferase reporter gene expression was then quantified as a marker of CNR1 promoter activity (Figure 6). Consistent with both our studies of Ca^2+^ imaging [10] and electrophysiology (this study), a familiar pattern of responses was observed. Thus, the stimulation of the CB_1_ receptors in naïve cells with the agonist WIN 55,212-2 (100 nM, 5 min) only marginally increased luciferase expression driven by the CNR1 promoter (Figure 6a). However, stimulation with ACh (1 µM, 15 min) produced a significant increase in luciferase expression, indicative of increased CNR1 promoter activity (Figure 6b). Strikingly, co-stimulation with ACh and WIN 55,212-2 stimulated CNR1 promoter activity to an even greater magnitude than ACh alone (Figure 6c). These data are therefore consistent with a model that the CNR1 promoter region is a component of the regulatory systems driving the interaction between the cannabinoid and ACh systems, by up-regulating the expression of the CB_1_ encoding CNR1 gene.

### 2.7. CREB Activation in SH-SY5Y Cells following Pre-Stimulation of M_3_ Receptors

**Figure 7:**
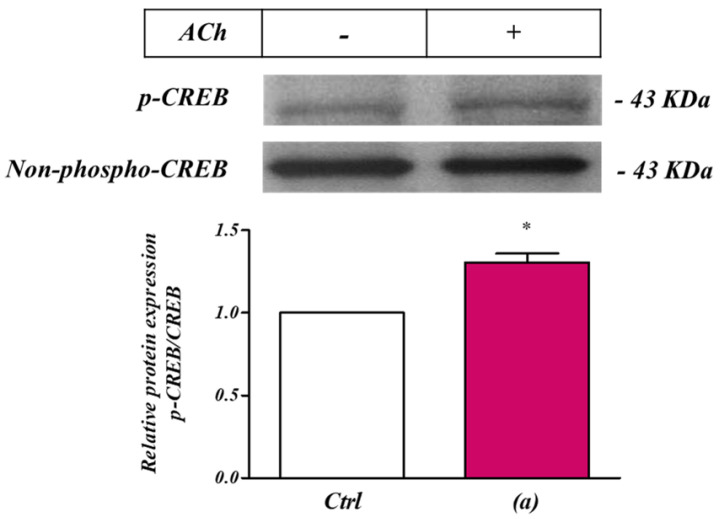
ACh ‘priming’ enhanced CREB activation in SH−SY5Y human neuroblastoma cells. Western blot analysis of the ACh priming effect on CREB expression level in SH−SY5Y neuroblastoma cells. 5 min pre-stimulation with ACh (ACh, 1 µM) induced a significant enhancement of CREB phosphorylation (**a**). * *p* < 0.05 vs. Ctrl. One−way analysis of variance, followed by Multiple Comparison test with Bonferroni’s correction. (*n* = 6).

If the promoter region of CNR1 is indeed activated by M_3_ receptor stimulation, it should also be possible to detect an increase in the activated form of CREB (phosphoCREB). Consistent with this, ACh administration produced a significant increase of phosphoCREB in SH-SY5Y cells (Figure 7a). This indicates that CREB is a downstream target of M_3_ receptors stimulation. 

## 3. Discussion

The disturbing symptoms of cannabis withdrawal have been shown to involve ACh signaling [26]. The current study aimed to determine whether there is cross-talk between the cellular signaling cascades governing cannabinoid receptor activation and ACh signaling, to explore a possible mechanism contributing to withdrawal symptoms. Collectively, our present and previous studies confirmed the presence of cross-talk between acetylcholine M_3_ receptors and cannabinoid CB_1_ receptors, at a cellular level through calcium signaling [6,7,10,27], and a direct interaction with the promoter region of the CNR1 gene. Overall, these findings support a model whereby M_3_ pre-activation evokes an increase in the cannabinoid-induced intracellular Ca^2+^ concentration, triggering CREB phosphorylation. There is also a concomitant, or possibly a directly consequential, rapid increase in both CB_1_ promoter activation and expression of the CB_1_ gene.

Both cannabinoid CB_1_ and acetylcholine M_3_ receptors are highly expressed in SH-SY5Y cells used in the present study, which is why they serve as good model for areas within the central nervous system, such as nucleus accumbens and amygdala, known to be linked to addiction [28,29,30,31]. Here, these receptors also play an important role in processes such as sensory and motor processing, sleep, nociception, mood, stress response, attention, arousal, memory, motivation, and reward [32,33]. There is also evidence for cholinergic involvement in the initiation of addictive processes. However, the mechanism by which M_3_ receptors affect the addiction induced by substance of abuse remains unclear [34,35]. Using a combination of electrophysiology, pharmacology, molecular biology, and bioinformatics, the current study supports a novel hypothesis whereby M_3_ receptors, via Ca^2+^ mediated regulation of the cross-talk, stimulate CB_1_ promoter activity, which may be involved in the complex events leading to the development of addictive behaviors or withdrawal syndromes, or both. 

To further elucidate the regulation of the cross-talk between receptors we first performed electrophysiological studies. Our results suggest that the ACh-primed increase in CB_1_ receptor-mediated inward current was a consequence of a series of coordinated events, ultimately leading to the activation of transcriptional mechanisms at the CNR1 locus that encodes the CB_1_ receptor. The abolition by 4-DAMP showed that M_3_ pre-stimulation was essential in the initiation of CB_1_ regulated depolarization. Our previous studies showed calcium mobilization acts as a major mediator of this cross-talk. Endoplasmic reticulum Ca^2+^ homeostasis and the stable interaction between SERCA2b and GPCRs, a receptor class which includes CB_1_ receptors, takes place during or soon after co-translation of the latter. This is a crucial event that enhances expression of GPCR receptors in a variety of cells [36]. Likewise, the stimulation of receptors that increase levels of the second messenger inositol trisphosphate (IP_3_), such as M_3_ receptors, cause the opening of the store-operated Ca^2+^ release-activated Ca^2+^ (CRAC) channels, activating components of the Ca^2+^ signaling pathway (STIM proteins, Orai channels, calmodulin). This is known to trigger transient transcriptional responses [37,38,39]. We therefore performed Western blotting, qPCR experiments and bioinformatics analysis, which indicated the pre-stimulation of M_3_ receptors in SH-SY5Y trigger the activation of a transcriptional mechanisms leading to new CB_1_ receptor expression, thereby suggesting the involvement of a CB_1_ promoter-centred autocrine loop. Our results indicate ACh pre-stimulation up-regulates CB_1_ receptors, by increasing CB_1_ gene promoter activity, mRNA transcript and protein. 

The role of transcription in the M_3_ receptor mediated regulation of CB_1_ receptors was particularly supported by the fact that CB_1_ receptor up-regulation was blocked by incubation with Act D, an antagonist of RNA polymerase II activity. The active involvement of the CNR1 promoter region was further supported by analysis of the CNR1 promoter on-line data repositories (UCSC genome database), which revealed active promoter markers such as histone modification marks (H3K4me3), binding of the core transcriptional apparatus (RNA polymerase II) and DNAaseI hypersensitivity binding sites are all present within the CNR1 promoter region in cell lines from which neuroblastomas such as SH-SY5Y cells are derived [40,41,42,43].

The active involvement of the CNRI promoter in the interaction of ACh and CB_1_ signaling with the expression of the CB_1_ gene was supported by the use of luciferase reporter plasmid containing the proximal promoter region sequence of CNR1 [24,44]. Individually, M_3_ receptor and CB_1_ agonisms both increase the activity of the CNR1 proximal promoter. However, combined M_3_ and CB_1_ agonism made the promoter much more highly active compared to their individual effects. These observations support the model that at least part of the molecular mechanism by which M_3_ stimulation enhances the electrophysiological response of CB_1_ activation is by activation of the CNR1 promoter. Since it is well established that release of intracellular Ca^2+^ can directly activate transcriptional factors, such as NFAT, NFkB, DREAM, CREB and *c-fos* [45,46,47,48,49], our previous observations suggest that intracellular Ca^2+^, mobilized due to M_3_ receptor stimulation, is likely to be the main second messenger involved in the CNR1 gene regulation. Indeed, the UCSC genome browser provides evidence that several of these transcription factors (i.e., NFkB, *c-fos*) bind to the CNR1 promoter region in neuroblastoma cell lines [24]. We also identified a number of potential transcriptional factor binding sites in the CNR1 promoter region. 

We focused our particular attention on CREB, since this transcription factor can be regulated by intracellular calcium concentration, which we have shown follows stimulation of M_3_ receptors in SH-SY5Y cells [10]. Our Western blotting showed a small but significant increase of the phosphorylated active form of CREB, indicating it may be activated by the stimulation of M_3_ receptors. This is consistent with studies in neurons showing intracellular Ca^2+^ signals can lead to CREB phosphorylation via activation of the CaM-kinase family of proteins [50]. In this context, in SH-SY5Y cells, muscarinic M_3_ receptor has been reported to regulate type 1 and type 8 adenylyl cyclase isoforms sensitive to Ca^2+^/calmodulin (CaM) or protein kinase C (PKC) [51]. In particular, it has been proposed that intracellular Ca^2+^ oscillations may be decoded by AC8 into parallel cAMP oscillations that in turn, could differentially be decoded by downstream targets (i.e., CREB) and potentially initiate events such as gene expression and cell differentiation [52].

Further, nuclear Ca^2+^ can be critical for activating regulatory pathways that control the timing and the nature of the CREB response. The hyper-excitable state of neurons in the nucleus accumbens after chronic exposure to drugs of abuse has been associated with changes in both Ca^2+^ homeostasis and CREB activation [10,25,53,54,55].

These observations, therefore, support a model whereby Ca^2+^ stimulation increases CREB phosphorylation. However, further studies are needed to determine if the effect of CREB phosphorylation is relayed through direct CNR1 promoter binding or result from an indirect interaction.

This current study is not the first to show a rapid modulation of CB_1_ receptor expression. A rapid increase of CB_1_ receptor expression has also been observed in human [56], other mammalian cell [57], and animal models [58], supporting the structure-specific plasticity induced by cannabinoid drugs indicated here. Interestingly, the rapid activation of the CNR1 gene observed in our experiments follows a very similar timeline to early gene activation in other studies [59,60,61].

**Figure 8:**
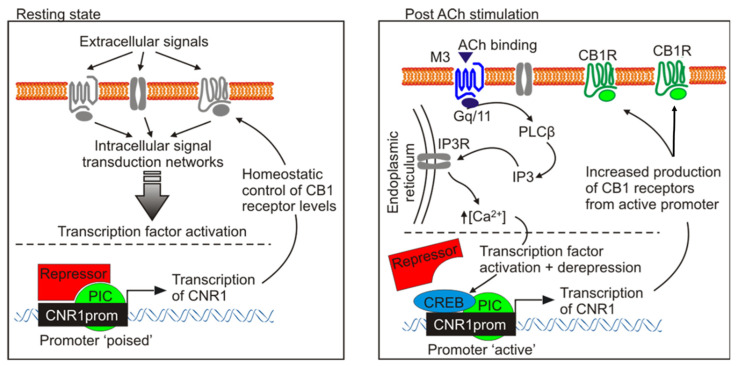
The proximal promoter region of CNR1 from a poised to an active state after ACh ‘priming’. Schematic representation of the proposed Gα_q/11_-Gα_i/o_ functional cross-talk, in SH-SY5Y neuroblastoma cells. At resting state (left panel) the CNR1 promoter is in a repressed state (poised promoter), allowing only the homeostatic control of CB_1_ receptors level. When primed with ACh, the M_3_ receptor signaling pathway is responsible for de-repressing the CNR1 promoter (unpoised promoter), that now becoming accessible to CREB, can actively promote active CNR1 gene transcription (right panel). PIC: promoter-bound preinitiation complex, which consists of RNA polymerase II and general transcription factors.

Altogether, our observations provide a mechanistic model to explain the functional cross-talk between M_3_ and CB_1_ receptors (Gα_q/11_-Gα_i/o_ cross-talk) that is responsible for the increased pharmacological response by ACh-priming (Figure 8). In this model, at a basal level, the stimulation of CB_1_ receptors produces a modest response, but the CNR1 promoter region is maintained in a state poised to respond if appropriately activated. The presence of an EZH2 binding site supports this situation. EZH2 is the functional enzymatic component of the Polycomb Repressive Complex (PRC2) that maintains gene promoter regions in a “poised” state, whereby release of PRC2 results in rapid transcriptional initiation [62,63]. Following M_3_ receptor activation, the increase in intracellular Ca^2+^ concentration primes the CNR1 promoter via the genetic machinery responsible for higher CB_1_ receptor protein expression. The increased insertion of these CB_1_ receptors into the cell membrane subsequently results in greater pharmacological responsiveness. Such increased responsiveness could underlie the symptoms of withdrawal.

In support of this model, the cross-talk mechanism we indicated by observations in the current proposal has been reported in the regulation of other genes. For example, a regulatory region that controls the BDNF gene in the hippocampus requires PKA and PKC pathways to interact appropriately [64]. Our model, therefore, could provide a significant step in the understanding of the cellular processes involving the neuronal responses to drugs of abuse such as cannabinoids. While most research to date has focused on synaptic plasticity to understand these processes, our current findings indicate a further mechanism. That is, changes in intrinsic neuronal excitability that are not synapse-specific, may play substantial roles in strongly modulating responses. Since a well-known feature of drug addiction is enhanced drug sensitivity, the observed Ca^2+^ mediated “homeostatic plasticity”, might be an important contributor to these cellular and behavioral adaptations involved in drugs of abuse phenomena [55].

Within the wider context of the burgeoning field of pharmacogenomics, our demonstration of the involvement of the CB_1_ promoter region in the observed crosstalk between the M_3_ and CB_1_ provides an opportunity to further explore the specific protein-DNA interactions involved. More importantly still, the identification of the role of the CB_1_ promoter in this mechanism will lead to an understanding of how genetic variation within the CB_1_ gene promoter, or even environmentally driven epigenetic modifications such as DNA-methylation, could lead to greater or lesser susceptibility to cannabinoid addiction and withdrawal.

## 4. Materials and Methods

### 4.1. Cell Cultures 

Human neuroblastoma SH-SY5Y cells (ATCC^®^ CRL-2266™, LGC Standards, Teddington, UK, passage 10–40) were grown as monolayers in Dulbecco’s Modified Eagle Medium (Sigma-Aldrich, St Louis, MO, USA), supplemented with 20% foetal bovine serum, 1 mM sodium pyruvate, 10 mM 4-(2-hydroxyethyl)-1-piperazineethanesulfonic acid (HEPES), 100 IU/mL penicillin and 100 µg/mL streptomycin. Cultures were seeded into 175 cm^2^ tissue culture flasks containing 25 mL of supplemented medium and maintained at 37 °C in 5% CO_2_/humidified air. Stock cultures were passaged at 1:10 weekly and media changed. For western blotting experiments, SH-5YSY cells were maintained as described above. For the electrophysiology experiments, cells were grown in monolayers in 6-well plates for 7 days before being used.

### 4.2. Electrophysiology 

The whole-cell configuration of the patch clamp technique [65] was used to record from cultured SH-SY5Y neuroblastoma cells at room temperature (20–23 °C). The pipettes had resistances of 3–7 MΩ and were filled with 140 mM CsCl, 1.1 mM EGTA, 2 mM MgCl_2_, 0.1 mM CaCl_2_, 2 mM ATP, 10 mM HEPES, and the pH of the internal patch pipette solution was adjusted to 7.2 by addition of Tris buffer. The patch pipettes were made from Pyrex borosilicate glass capillaries (Plowden and Thompson Ltd., Dial Glass Works, Stourbridge, UK) using a two-stage vertical microelectrode puller (Model 730, David Kopf Instruments, Tujunca, CA, USA). During experiments, the cells were maintained in a 35 mm diameter culture dish with extracellular recording buffer medium: 152 mM NaCl, 5 mM KCl, 1 mM MgCl_2_, 2 mM CaCl_2_, 10 mM HEPES and 10 mM Glucose. The pH was adjusted to 7.4 by addition of NaOH. The osmolarity of the external recording medium and internal patch pipette solutions were adjusted to 320 and 310 mOsm, respectively, with sucrose. Frozen external medium solutions were freshly defrosted and CaCl_2_ was added as required. In some cases, CaCl_2_ was substituted with BaCl_2_ (2 mM) in the extracellular recording or internal patch pipette solution, as the charge carrier for calcium channel currents. In some experiments CaCl_2_ was omitted and the concentration of the calcium chelator EGTA was raised to 20 mM to test for calcium-dependent conductance. The cells were viewed using a Diaphot-TMD (Nikon Europe B.V. Amstelveen, The Netherlands) inverted microscope at x200 magnification. A ground electrode in a KCl bath was connected to the adjacent recording bath by a KCl-agar bridge. Patched cells were voltage clamped with an Axoclamp-2A amplifier (Axon Instruments, Molecular Devices, Wokingham, UK) at a sampling rate of 15–25 kHz in the discontinuous single electrode voltage clamp mode, and initially held at −70 mV. Voltage steps were triggered, and the step duration controlled, by a D4030 pulse generator (Digitimiter, Welwyn Garden City, UK). Data were acquired with Scope 3.6.11 software (PowerLab, AD Instruments Ltd, Oxford, UK). Antagonists were bath-applied (M_3_ antagonist, 4-DAMP; CB_1_ antagonist/inverse agonist, AM251) while agonists were applied focally to the surface of the cells using a low-pressure (~7K Pa) ejection (ACh, WIN; PDES-2DX-LA, npi electronic GmbH, Tamm, Germany) from micropipettes with a diameter of 10 µm, with the pipette tip placed approximately 100 µm from the cell from which responses were being recorded.

### 4.3. Western Immunoblotting

Samples (20 µg total protein, quantified by Bradford Protein Assay, Bio-Rad©, Watford, UK) for western blotting were separated by 10% SDS-PAGE using the BioRad© minigel system. Proteins were electrotransferred to nitrocellulose membranes using the BioRad© semi-dry blotter apparatus according to manufacturer’s instructions. Following electrotransfer, the membranes were blocked for non-specific binding at room temperature (20 °C) for 1 h in Tris-buffered saline (50 mM Tris-HCl, 150 mM NaCl, pH 7.6) containing 0.1% Tween-20 (TBS-T), supplemented with 5% non-fat milk. After blocking, membranes were washed in TBS-T, and incubated overnight at 4 °C with either rabbit polyclonal pCREB-1 (ser 133)-specific antibody (1:200 dilution in 5% milk/TBS-T, sc-7978, Santa Cruz Biotechnology, Santa Cruz, CA, USA) or rabbit polyclonal CREB-1 (C-21)-specific antibody (1:200 dilution in 5% milk/TBS-T, sc-186, Santa Cruz Biotechnology, Santa Cruz, CA, USA) or rabbit polyclonal CB_1_ (H-150)-specific antibody (1:250 dilution in 5% milk/TBS-T, sc-20754, Santa Cruz Biotechnology, Santa Cruz, CA, USA). Primary antibodies were detected for 1 h at room temperature with a secondary antibody (Donkey anti-rabbit, sc-2313, 1:1000 dilution, Santa Cruz Biotechnology, Santa Cruz, CA, USA) conjugated to horseradish peroxidase. This was followed by chemo-luminescence detection using ECL-plus reagent and exposure to HyperFilm^TM^ (Amersham Life Science GE Healthcare Life Sciences, Little Chalfont, UK). To ensure equal protein loading across the gel, membranes were submerged in stripping buffer (62.5 mM Tris/HCL, 100 mM 2-mercaptoethanol, 2% (*w*/*v*) SDS, pH 6.7), and incubated at 50 °C for 40 min, and re-probed with a goat polyclonal GADPH antibody (1:500 dilution in 5% milk-TBS-T, sc20357, Santa Cruz Biotechnology, Santa Cruz, CA, USA), followed by detection of the primary antibody with donkey anti-goat secondary antibody (sc-2020, 1:1000 dilution, Santa Cruz Biotechnology, Santa Cruz, CA, USA). The band intensity on the Western blots was quantified by densitometry with Image-Pro Plus 6.0 software. (Media Cybernetics, Inc., Rockville, MD, USA).

### 4.4. Quantitative Reverse-Transcription PCR

A two-step method for the quantification of CNR1 transcript levels was used. SH-SY5Y neuroblastoma cells total RNA was extracted using ReliaPrep RNA cell miniprep system (Promega, Southampton, UK) in accordance with manufacturer’s recommendations. A DNaseI incubation step was included in the protocol. Purified RNA concentrations were measured on a spectrophotometer (Nanodrop, ThermoFisher Scientific, Loughborough, UK) and aliquots of purified RNA were made to a final concentration of 60 ng/µL with RNase-free H_2_O. RNA was reverse transcribed to cDNA using the High-Capacity cDNA reverse transcription kit (Applied Biosciences, ThermoFisher Scientific, Loughborough, UK) using random primers, 600 ng of RNA and the following thermocycling conditions: 10 min at 25 °C then 120 min at 37 °C and finally 5 min at 85 °C. cDNA samples were diluted to a final concentration of 10 ng/µL with pure water after the completion of reverse transcription. Quantitative PCR was conducted with 2× SYBR green master mix (Roche, Welwyn Garden City, UK) in a Light Cycler 480 (Roche), with all data being analyzed on Light Cycler 480 software (Roche, Welwyn Garden City, UK). Master Mix reaction solutions were prepared containing 1× SYBR green, 500 nM final concentration CNR1 primers and TBP primers as a reference gene [66]. Master mixes were divided 18 µL/well in 384-well plates and cDNA samples were loaded at 2 µL/well in triplicate. Standard curves were produced for both primer pairs using a 5-fold serial dilution of one of the cDNA samples to provide a range from undiluted to 125 times diluted. cDNA samples for the production of a standard curve were loaded at 2 µL/well in duplicate. Standard curves from CNR1 primers and TBP primers were used to calculate primer efficiency values specific to both the primers being utilized and the sample being measured. Quantitative PCR thermocycling conditions were as follows: 5 min at 95 °C, 45 cycles of: 10 s at 95 °C, 10 s at 56 °C, 10 s at 72 °C, followed by a melting curve analysis using: 5 s at 95 °C, 1 min at 65 °C, 97 °C continuous. 

Quantitative PCR data was analyzed using advanced relative quantification strategy implementing assay-specific primer efficiencies. Primers for qPCR were:

CNR1 forward: 5′ CCTACCTGATGTTCTGGAT 3′
CNR1 reverse: 5′ TGGATGATGATGCTCTTCT 3′
TBP forward: 5′ TTAGTCCAATGATGCCTTATG 3′
TBP reverse: 5′ CTGCCTTTGTTGCTCTTC 3′

### 4.5. Bioinformatics

In silico bioinformatic analysis of the CNR1 locus and CNR1 proximal promoter region were conducted using the ECR browser, rVista and the Transfac professional (v 10.2) transcription factor binding site algorithm hosted by the ECR browser [67]. CREB-binding sites within the human CNR1 promoter sequence were searched for using a matrix similarity value of 0.85 or greater. Further bioinformatics analysis of the CNR1 locus and CNR1 proximal promoter region was conducted using data hosted by the UCSC browser [68]. CNR1 locus annotation derived from RefSeq and Aceview. Polyadenylation sites by Gencode version 19 and consensus coding DNA sequences (CDS) came from the collaborative project between NCBI, EBI, HGNC, MGI and WTS. DNaseI hypersensitivity plots, ChIP-seq data on transcription factor binding, histone modifications and chromatin state segmentation data were collected from ENCODE [69] project data hosted by the UCSC browser.

### 4.6. Cloning

The CNR1 proximal promoter fragment was amplified from human placental DNA using the following primers: forward 5′ CGCAGCCAGGTAGCGAACG 3′ and reverse 5′ TTTCGTTCTAGCGGACAAC 3′. Primers were designed to amplify the negative genomic DNA strand since CNR1 is transcribed from this strand. Following PCR amplification, the specific CNR1 proximal promoter amplicon was cloned into a linearized pGEM–T Easy vector using T4 DNA ligase (Promega, Southampton, UK). The CNR1 proximal promoter fragment was then cloned into pGL4.23 (Promega, Southampton, UK) by digesting both vector and insert plasmids with EcoRI and SacI to form pCNR1prom-Luc. High quality pCNR1prom reporter plasmid was purified from transformed *E. coli* using an endotoxin free maxi prep kit (Qiagen, Manchester, UK).

### 4.7. Luciferase Reporter Assays

Cells (≈1 × 10^4^) were plated onto 24-well plates 24 h before transfection with Transfast (Promega, Southampton, UK). Transfections were optimized according to the manufacturer’s instructions. In brief, reporter plasmid (the CNR1prom-Luc and pGL4.74 (renilla luciferase Promega), 0.5 μg per well) was mixed with serum-free cell medium (200 μL per well), and Transfast was added at a ratio of 2:1 (3 μL per well). Cells were then washed twice with PBS, and DNA medium mixture was added for 1 h, after which 1 mL of relevant cell medium containing 10% FBS was added. Following transfection and treatment of SH-SY5Y neuroblastoma cells, culture plates were placed on ice and washed with cold PBS twice followed by 20 min incubation at room temperature with 150 µL of 1× passive lysis buffer (Promega, Southampton, UK). Cell lysates were collected in pre-cooled micro tubes and centrifuged at 4000× *g* for 5 min at 4 °C. Cleared lysates were then loaded (20 µL/well) into a 96-well luminometer plate (Nunc, Roskilde, Denmark) in duplicate. Firefly luciferase and *Renilla* luciferase intensities were measured on GloMax^®^ 96 Microplate Luminometer (Promega, Southampton, UK) using the Dual-Luciferase reporter assay system in accordance with manufacturer’s instructions. Reporter assay data were analyzed by calculating the ratio between firefly luciferase and *Renilla* luciferase intensities to control for differences in transfection efficiency. Subsequently, percentage change was calculated for treated samples as compared with control samples. 

### 4.8. Statistical Analysis

The data and statistical analysis comply with the recommendations on experimental design and analysis in pharmacology [70]. Specifically, all averaged data sets are expressed as the mean ± S.E.M of *n* independent experiments. Statistical analysis was performed by either Student’s unpaired *t*-test or one-way analysis of variance (ANOVA) followed by Bonferroni’s Multiple Comparison post hoc test as appropriate (GraphPad Prism, San Diego, CA, USA).

## 5. Conclusions

This study revealed for the first time a functional interaction between CB_1_ and M_3_ receptors, that through the intracellular Ca^2+^ mobilization pathway, is responsible for a rapid transcription of the CNR1 gene. Our findings clearly demonstrate how the physiological responses due to CB_1_ receptor stimulation could be controlled and modulated by the cholinergic system. This newly described mechanism suggests the possibility of targeting the cannabinoid system from a different angle, with the cholinergic system playing a central role. In this contest, considering the cholinergic system the primary target, its activation could modulate cannabinoid system pharmacological responses, as well as limiting the manifestation of the unwanted side effects (withdrawal and addiction).

## Data Availability

Data is contained within the article.
